# A132 PREDICTIVE VALUE OF ENDOSCOPIC EVALUATION IN EOSINOPHILIC ESOPHAGITIS

**DOI:** 10.1093/jcag/gwae059.132

**Published:** 2025-02-10

**Authors:** I Stukalin, M Kota, N Gonsalves, M Gupta, C Ma

**Affiliations:** Gastroenterology, University of Calgary, Calgary, AB, Canada; Gastroenterology, Northwestern University Feinberg School of Medicine, Chicago, IL; Gastroenterology, Northwestern University Feinberg School of Medicine, Chicago, IL; Gastroenterology, University of Calgary, Calgary, AB, Canada; Gastroenterology, University of Calgary, Calgary, AB, Canada

## Abstract

**Background:**

Clinical trials for eosinophilic esophagitis (EoE) currently use the peak eosinophil count (PEC) and patient-reported outcomes as coprimary endpoints for determining therapeutic efficacy. Although the PEC reflects histological activity in EoE, it has not been consistently correlated with symptoms nor endoscopic appearance and the prognostic value of endoscopy relative to the PEC is unclear.

**Aims:**

We evaluated the Endoscopic Reference Score for EoE (EREFS) as a predictor of clinically relevant endpoints.

**Methods:**

We evaluated a retrospective prevalent cohort of adults (>18) with EoE undergoing upper endoscopy between 2010 and 2023 from two tertiary care institutions. The EREFS total score, individual components, inflammatory and fibrostenotic subscores were assessed. The primary endpoint was a composite outcome of food bolus impaction (FBI), requirement for esophageal dilation, and hospitalization for EoE within 12 months of the baseline endoscopy. Logistic regression was used to assess the association between EREFS and the composite endpoint.

**Results:**

A total of 350 patients were included (225 [64.3%] male, mean age at presentation 35.0 years [SD 13.6], 299 [85.4%] presenting with predominantly dysphagia). Mean EREFS at baseline was 4.3 (SD 1.8), with a mean inflammatory subscore of 2.3 (SD 1.3) and fibrostenotic subscore of 2.0 (SD 1.0). Most patients had a baseline stricture (269/350, 76.9%) and 135 (38.6%) underwent dilation at baseline to a mean diameter of 14.3 mm (SD 2.4). The mean baseline PEC was 44.3 eos/HPF (SD 35.1). Baseline EREFS was significantly associated with the composite primary outcome (OR 1.32 [95% CI: 1.16, 1.50], p<0.001) whereas the PEC was not associated (OR 1.03 [95% CI: 0.97, 1.10], p=0.30 for each 10 eos/HPF increase). The baseline fibrostenotic EREFS (OR 2.04 [95% CI: 1.58, 2.64], p<0.001) and presence of stricture (OR 6.12 [95% CI: 3.28, 11.43], p<0.001) were significantly associated with the composite primary endpoint, even when patients with baseline dilation were excluded.

**Conclusions:**

The fibrostenotic EREFS subscore is significantly associated with the requirement for future dilation and food bolus impaction, with prognostic value beyond that of the PEC alone.

**Funding Agencies:**

None

